# Post resuscitation care – some words of caution and a call for action

**DOI:** 10.1186/s13049-015-0167-2

**Published:** 2015-11-04

**Authors:** Eldar Søreide, Alf Inge Larsen

**Affiliations:** Department of Anaesthesiology and Intensive Care, Stavanger University Hospital, Stavanger, Norway; Department of Clinical Medicine, University of Bergen, Bergen, Norway; Network for Medical Sciences, University of Stavanger, Stavanger, Norway; Department of Cardiology, Stavanger University Hospital, Stavanger, Norway; Department of Clinical Science, University of Bergen, Bergen, Norway

**Keywords:** Out-of-hospital cardiac arrest, Resuscitation, Intensive care, Critical care, Prognostication, Target temperature management, Acute myocardial infarction, Coronary angiography, Percutaneous coronary intervention, Acute cardiac care, Survey

## Abstract

This fall the European Resuscitation Council (ERC) and the European Cardiology Society (ESC) publish updated post resuscitation care guidelines. For these guidelines to have an impact they must be implemented into daily clinical practice. Newer studies imply that differences in hospital care explain much of the observed differences in survival after out-of-hospital cardiac arrest. A recent Nordic (Denmark, Finland, Iceland, Norway, Sweden) survey suggests worrisome variations in post resuscitation care provided and should urge us all to act in the coming years. One important step will be to build up resuscitation systems with integrated cardiac arrest centres in all the 5 Nordic countries and benchmark process of care, financial implications and survival.

## Introduction

Every day unconscious survivors from an out-of-hospital cardiac arrest (OHCA) fight for their lives in Intensive Care Units (ICU) throughout Europe. Despite all the efforts, including targeted temperature management (TTM) and invasive coronary interventions, a significant number of them die before hospital discharge. A recent Swedish single-centre study found the cause of death to be cardiac in 16 % of these patients, with another 9 % dying from sepsis, multi organ failure or trauma [1]. However, anoxic brain injury remains the big killer with 75 % of deaths being linked to the patient never waking up and withdrawal of care [1]. Another European study has corroborated these findings [2].

**Table 1:** How to improve Nordic post resuscitation care

1.	Seek multi-speciality consensus on what constitute best practice
2.	Support national implementation of regional resuscitation systems with integrated cardiac arrest centres
3.	Audit results in terms of process of care and outcomes
4.	Benchmark, revise and implement the changes needed on a national basis

Recently, this *Journal* published a Nordic (Denmark, Finland, Iceland, Norway, Sweden) survey of post resuscitation care in OHCA patients [3]. The main finding was a huge and unexplained difference in care provided between hospitals and countries. Assuming ICU and emergency physicians and cardiologists read the same medical science and follow the same international clinical guidelines [4–6], this finding is intriguing and warrants some reflection.

It has been pointed out that lack of local implementation may be the culprit for much of the noted differences in survival rates after OHCA [7]. With updated European guidelines for post resuscitation care [8, 9] just being published, we think the study by Saarinin et al. [3] is very pertinent and should act as a call for action. Simultaneously, we think a word of caution is needed. Do we have the appropriate scientific evidence for all the contemporary guidelines, or are they just systematic presentations of expert opinion? Do we know which part of the guidelines that works in real life and makes a difference? Last but not least, does the present survey [3] reflect modern post resuscitation care in the Nordic countries? If the answer is yes to the latter question, there is an obvious need for a Nordic consensus process regarding specialized resuscitation systems with integrated cardiac arrest centres.

### Post resuscitation care guidelines

Since 2005 the European Resuscitation Council (ERC) guidelines have focused on three main aspects of post resuscitation care; target temperature management (TTM) - also called therapeutic hypothermia-, immediate coronary angiography (CAG) with percutaneous coronary intervention (PCI) when appropriate, and proper prognostication before withdrawal of care. To secure high quality care, the guidelines also focus on the importance of regionalized care and specialised cardiac arrest centres [4, 5, 8]. Comparing the concept of cardiac arrest centres to already well-established trauma, stroke and primary PCI for STEMI (“heart attacks”) centres make sense. They all involve proper patient transfer logistics with adequate communication net-works and strict time limits. Patients with a cerebrovascular event are now being canalized to centres with on-site capability of 24/7 high-speed perfusion CT supported neurological diagnosis and pharmaco-mechanical intervention if systemic thrombolysis fails [9]. The costs are large, but the consequences of a fulminant cerebral stroke in an otherwise healthy individual are catastrophic both for the patients, the family and the society.

The algorithms for modern stroke treatment are based on the successful history of primary PCI for ST-elevation myocardial infarction (STEMI) [10–12]. In STEMI OHCA cases there is also an indication for CAG [13]. Approximately 60 % of these patients have a flow limiting stenosis [14]. Even without ST-elevation at least 25 % of the patients turn out to have a coronary occlusion [15, 16]. The numbers of reports supporting early revascularization in OHCA patients with transmural ischemia indicating a large area of myocardium at risk are building up. In Europe, CAG and subsequently PCI within 2 h in OHCA survivors with STEMI have a 1A indication [13]. Recent North-American guidelines also support this aggressive approach [17]. The consequences of immediate revascularization on malignant arrhythmias and hemodynamic instability are obvious. The long-term effects of preserving a well-functioning left ventricle are even more important [18, 19]. Survivors with large not re-perfused myocardial infarctions, both STEMI and non-STEMIs, have a poor prognosis (both 30 days and long term) due to reduced left ventricular ejection fraction with concomitant serious heart failure (HF) and malignant arrhythmias. Modern therapy that prevents and ameliorates this development is associated with improved prognosis [20].

In the current ESA 2015 guidelines, immediate CAG in survivors of OHCA with a non-STEMI is given a 1C recommendation [13]. A 1C recommendation means that it is based on a consensus of experts as randomized studies in this special population have not been performed. However, one such study is underway in Sweden [21]. Rapid echocardiography is an important part of the acute cardiac care offered OHCA survivors. Some invasive cardiologist thinks that OHCA patients without ST segment elevation and with a normal left ventricle, as assessed with echocardiography, may be offered CAG at a later point of time if stable. In patients with cardiogenic shock immediate revascularization might be the only option to stabilize hemodynamics [22].

Present logistic networks for treating STEMI patients in a timely fashion have been proven to yield the largest survival benefits in modern cardiology. There is no reason not to offer comatose surviving OHCA patients with acute coronary syndrome the same proper treatment. Cardiac arrest centres must work in tight conjunction with the prehospital emergency medical services (EMS) system. The success in terms of improved long-term survival is not linked to the specialized hospital or ambulance service alone, but the integrated high quality care provided throughout the Chain of Survival [23] from telephone-assisted bystander CPR before ambulance arrival to ICD implantation, if necessary, before hospital discharge.

Importantly, appropriate prognostication and withdrawal of care is also a key issue in post resuscitation care. If the prognostication is done prematurely and based on inappropriate tests, the result will be self-fulfilling prophecies and poor survival rates. Therefore, European intensive care and resuscitation bodies [24] have produced evidence based guidelines for neuroprognostication. Little is known how they are implemented into clinical practice. Studies so far have not been very encouraging [25].

### Post resuscitation care in the Nordic countries

Saarinen et al. [3] found an overall large variation in the approach to OHCA patients in the Nordic countries. While almost 80 % of the respondents in Denmark and Norway reported that they used a predefined protocol, the corresponding number for Finland and Sweden was below 50 %. The survey does not tell us why this is so, but we perceive a large trust in care based on physician discretion. When looking at specific post resuscitation therapies, the authors uncovered the same pattern. While almost 70 % of Norwegian ICUs offer TTM to all unconscious OHCA survivors admitted, the corresponding number for Finland and Sweden are 20 and 25 %, correspondingly. The great difference in care provided, however, starts earlier. Of the around 60 % ICUs reporting to have predefined protocols to define which patients to admit, unreliable negative predictors of bad outcomes like age, pre-arrest health, low-flow time, initial rhythm and cause of OHCA were all in use [3].

Most ICUs in the survey used TTM with 33° C as target temperature, also after the so-called TTM trial [26] comparing 33 to 36° C for 24 h did not identify a significant difference in outcomes between those two TTM approaches.

Post OHCA CAG and PCI practices also varied a lot from country to country (ref). While a rather active approach towards acute CAG and PCI prevailed in Sweden and Norway with more than 50 % of the hospitals outinely offering emergency CAG and PCI to OHCA survivors, the corresponding number for Finland was 13 %.

In terms of prognostication and outcome, the finding that so many unconscious OHCA victims do not get admitted to an ICU for mechanical ventilation and general neuro-intensive care in the first place [3] should cause concern. Further, that prognostication is based on clinical exam alone, or in combination with EEG, is also worrisome. Especially, as a large proportion perform the prognostication quite early after the event [3]. Only 1 of 3 ICU made use of somatosensory evoked potential (SSEP), a method found to be of great value [24]. The unsophisticated approach to prognostication contrast with recent recommendations [24] but fits in with a similar survey performed in 2008 [27]. The chance of self-fulfilling prophecies for a bad prognosis appears to be prominent in Nordic ICUs.

The overall impression from the survey by Saarinen et al. [3] is that very little have actually changed over the last 10 years in Nordic post resuscitation care. Unfortunately, the survey did not contain questions about cardiac arrest centres and integrated EMS systems.

In a recent Norwegian study [28], clinical practice and outcomes in three university hospitals fulfilling the criteria for cardiac arrest centre were compared. The overall survival in unconscious OHCA survivors of all age groups and causes was almost identical and quite high (42 %). This study does not prove the concept of cardiac arrest centres, but may give some indications that an aggressive therapeutic approach is worthwhile [29, 30]. More recent studies from Denmark have substantiated the concept that larger and specialized cardiac arrest centres do save more lives [31, 32]. Only 30 % of the reporting ICUs in the present survey [3] treated ≥ 40 OHCA patients annually. Hence, building a case for regionalized care and cardiac arrest centres in the Nordic countries should be quite easy.

### Some words of caution and a call for action

Given its many methodological limitations [33, 34], how much can we extract about the present status of post resuscitation care in the Nordic countries from the present survey? The response rate was far from optimal and we do not know how the questionnaire was developed and to what extent language misunderstandings may have affected the answers provided. What constitute ICU care in this population may have been defined differently. We do not for sure that the answers given by the respondents [3] reflect current practice in their ICU. The fact that most respondents were senior ICU physician, however, means that this was probably the case. Further, does the non-adherence to current post resuscitation care guidelines revealed in this survey reflect worrisome sub-standard care or just that nobody really knows who benefit from modern post resuscitation care? Although the latter may partly be through, we think a certain hint of therapeutic inertia and concealed financial concerns is also present in the given answers.

For those obsessed with evidence based medicine and waiting for the next perfect randomized controlled trial to delineate every aspect of post resuscitation care, we also think a word of caution is needed. Post resuscitation care is a complex intervention and as such may be better served by bench-marking care provided in centres with good outcomes to those lagging behind [31, 32]. Using registry data to monitor OHCA outcomes allow a continuous quality improvement process [23]. Although not highly rated in academic medicine, we think combining the best clinical evidence available with expert opinion is the way to go to improve the current situation in the Nordic countries (Table 1).

## Conclusions

This fall ERC [8] and the European Cardiology Society [13] publish their new post resuscitation clinical guidelines. For these guidelines to have an impact, we need to make sure they are implemented [7]. Newer studies from all over the world, including Nordic countries, have shown that hospital care after OHCA does matter [31, 32, 35, 36] and may explain large proportions of the difference in outcomes noted. A recent Nordic survey [3] indicate a worrisome variation in post resuscitation care provided and should urge as all to act in the coming years. One important step will be to build up resuscitation systems with integrated cardiac arrest centres in the 5 Nordic countries and benchmark process of care, financial implications and survival outcomes in the years to come. The survey by Saarinin et al. [3] should act as a candid call for immediate action.
